# Abbreviated MRI protocol for colorectal liver metastases: How the radiologist could work in pre surgical setting

**DOI:** 10.1371/journal.pone.0241431

**Published:** 2020-11-19

**Authors:** Vincenza Granata, Roberta Fusco, Antonio Avallone, Antonino Cassata, Raffaele Palaia, Paolo Delrio, Roberta Grassi, Fabiana Tatangelo, Giulia Grazzini, Francesco Izzo, Antonella Petrillo

**Affiliations:** 1 Radiology Division, “Istituto Nazionale Tumori IRCCS Fondazione Pascale – IRCCS di Napoli”, Naples, Italy; 2 Gastrointestinal Oncology Division, “Istituto Nazionale Tumori IRCCS Fondazione Pascale – IRCCS di Napoli”, Naples, Italy; 3 Hepatobiliary Surgical Oncology Division, “Istituto Nazionale Tumori IRCCS Fondazione Pascale – IRCCS di Napoli”, Naples, Italy; 4 Division of Gastrointestinal Surgical Oncology, “Istituto Nazionale Tumori IRCCS Fondazione Pascale – IRCCS di Napoli”, Naples, Italy; 5 Division of Radiology, University of Campania Luigi Vanvitelli, Naples, Italy; 6 Division of Pathology, “Istituto Nazionale Tumori IRCCS Fondazione Pascale – IRCCS di Napoli”, Naples, Italy; 7 Division of Radiology, “Azienda Ospedaliera Universitaria Careggi”, Florence, Italy; Humanitas Clinical and Research Center - IRRCS, ITALY

## Abstract

**Background:**

MRI is the most reliable imaging modality that allows to assess liver metastases. Our purpose is to compare the per-lesion and per-patient detection rate of gadoxetic acid-(Gd-EOB) enhanced liver MRI and fast MR protocol including Diffusion Weighted Imaging (DWI) and T2-W Fat Suppression sequence in the detection of liver metastasis in pre surgical setting.

**Methods:**

One hundred and eight patients with pathologically proven liver metastases (756 liver metastases) underwent Gd-EOBMRI were enrolled in this study. Three radiologist independently graded the presence of liver lesions on a five-point confidence scale assessed only abbreviated protocol (DWI and sampling perfection with application-optimized contrasts using different flip angle evolution (SPACE) fat suppressed sequence) and after an interval of more than 2 weeks the conventional study (all acquired sequences). Per-lesion and per-patient detection rate of metastases were calculated. Weighted к values were used to evaluate inter-reader agreement of the confidence scale regarding the presence of the lesion.

**Results:**

MRI detected 732 liver metastases. All lesions were identified both by conventional study as by abbreviated protocol. In terms of per-lesion detection rate of liver metastasis, all three readers had higher detection rate both with abbreviated protocol and with standard protocol with Gd-EOB (96.8% [732 of 756] vs. 96.5% [730 of 756] for reader 1; 95.8% [725 of 756] vs. 95.2% [720 of 756] for reader 2; 96.5% [730 of 756] vs. 96.5% [730 of 756] for reader 3). Inter-reader agreement of lesions detection rate between the three radiologists was excellent (k range, 0.86–0.98) both for Gd-EOB MRI and for Fast protocol (k range, 0.89–0.99).

**Conclusion:**

Abbreviated protocol showed the same detection rate than conventional study in detection of liver metastases.

## Introduction

Imaging is an important tool in the management of patients with liver metastases by helping enumerate the number and sites of lesions, assessing the resectability, evaluating the response to treatment (systemic or ablative therapies), and detecting drug toxicities [1–4]. Although multidetector computed tomography (MDCT) is routinely used for primary staging and disease surveillance, Magnetic Resonance imaging (MRI) is a valuable diagnostic technique in oncologic setting, since this tool provides morphological and functional data [5–8]. Functional data, extracted by diffusion weighted imaging (DWI) and dynamic contrast-enhanced (DCE)-MRI, allow a proper detection and characterization of focal liver lesions [5–8]. Oncology is a major field of application of DWI, especially in the detection and characterization of liver metastases [9–11]. The analysis of DW data can be done qualitatively and quantitatively, through the apparent diffusion coefficient (ADC) map, which is the graphical representation of the ratio of DW signal intensities and its measurements and it may discriminate between benign and malignant lesions. The ADC values are related to the sequence acquisition protocol and suffer from a lack of reproducibility, especially in respiratory triggering techniques, nodules of left lobe, smaller size and lesion heterogeneity [12,13]. The ADC values for metastases show a significant overlap between ADC values of benign hepatocellular lesions and other malignant lesions [14]. Lesion characterization should therefore be done considering also morphological and functional data obtained by T2-W and T1-W sequences and dynamic studies [15,16]. Various liver-specific contrast media (cm) have been developed to improve the detection and characterization of hepatic lesions. Gadobenate dimeglumine (Gd-BOPTA) and gadolinium ethoxybenzyl diethylenetriamine pentaacetic acid (Gd-EOB-DTPA) can be injected as an intravenous bolus, providing data about lesion vascularity in the different phases of contrast circulation. In addition, functional data can be obtained in the delayed, hepatobiliary phase [17–20]. Although the Gadolinium chelates (GBCAs) are safe, adverse reactions induced by their administration have been reported; moreover several patients cannot be administered GBCAs. [21–25]. Although contrast medium is a useful tool in the characterizing setting, however in pre surgical setting after conversion treatment, the radiologist’s role is identifying residual metastases in order to assess the resectability.

The aim of this study was to compare the per-lesion and per-patient detection rate of gadoxetic acid-enhanced liver (Gd-EOB) MRI versus abbreviated protocol (only DWI and T2-W fat suppressed (FS) sequences) in the detection of liver metastasis, using liver resection as the reference standard.

## Methods

### Study population

Institutional review board of National Cancer Institute of Naples approved this retrospective study, and the patient’s informed consent requirement has been waived. From January 2015 to September 2019 we selected 124 patients with liver colorectal metastasis (mCRC), who underwent liver resection. The inclusion criteria for the study population were as follows: *(a)* patients with pathologically-proven mCRC; *(b)* patients who had subjects to Gd-EOB MRI within 1 month to surgical resection; *(c)* patients who had less than a 1-month between radiological and pathological diagnosis; and *(d)* accessibility of diagnostic quality pictures of the cut sections of the resected specimens. The exclusion criteria were as follows: no accessible or absent Gd-EOB MR study.

**Fig 1:**
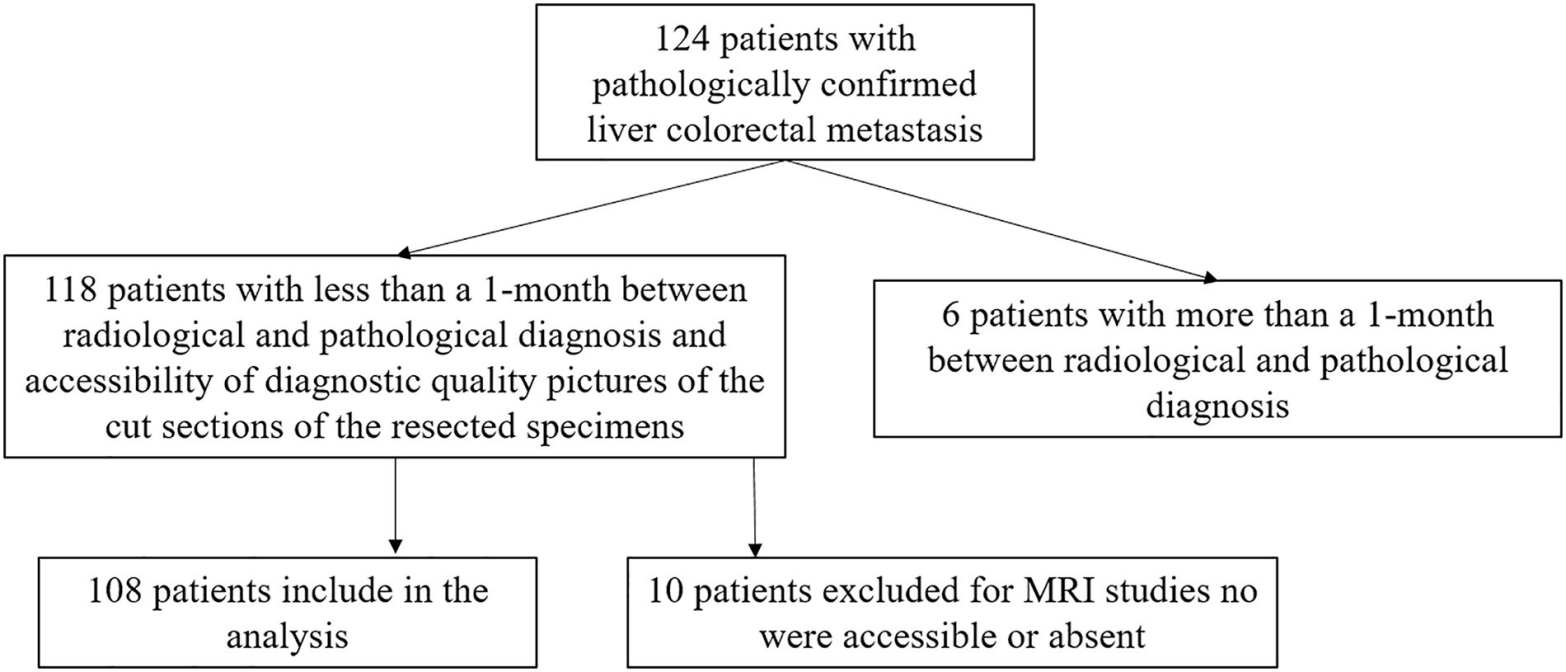
Flow chart of included and excluded patients.

Among 124 patients, 118 with mCRC confirmed at pathological analysis satisfied the inclusion criteria because 6 patients had more than a 1-month between radiological and pathological diagnosis. Among these118 patients, 10 were excluded because MRI studies no were accessible or absent (see Fig 1). Finally, 756 pathologically proven lesions (median 7, range 1–9 per patient), diagnosed as mCRC in 108 included patients [56 women-52 men; median age, 62 years; range, 35–78 years) comprised our study population. Characteristics of the 108 patients are summarized in Table 1. The correspondence between pathologically proven lesion and detected lesion by MRI was verified by means of surgical report and pathological report.

**Table 1:** Data of the patient population.

	mCRC patients (no. = 108)
***Demographics***	
Gender	Men 52 (48.1%)
	Women 56 (51.8%)
Age	Median, 61.1 years
	Range, 35–78 years
***Primary cancer site***	
Colon	72 (66.7%)
Rectum	36 (33.3%)
***History of chemotherapy***	108 (100%)
***Liver metastases***	
Number	756
	median 7 per patient
	range 1–9 per patient
Largest diameter	median 28 mm
	range 8–57 mm

### Lesion confirmation: Reference standard

A pathologist, specialized in liver, performed histopathologic analysis of resected specimens. One hundred and eight patients with 756 pathologically proven lesions who underwent surgical resection (median tumor size, 28 mm; range 8–57 mm) constituted the study group. Lesion confirmation was based on the pathologic diagnosis of surgically resected liver specimens. The resected specimens were processed and then sectioned with a 5-mm slice thickness. All tumor samples were marked with hematoxylin and eosin coloration. Immunohistochemistry stains were obtained to verify the intestinal origin of the lesions. The panel of immunohistochemical markers included cytokeratin 7, cytokeratin 20, and CDX2. The histopathological report included the pushing or infiltrating growth and the presence or absence of tumor budding and/or fibrosis and necrosis.

### MR imaging protocol

MR studies was performed with a 1.5T scanner (Magnetom Symphony, with Total Imaging Matrix Package, Siemens, Erlangen, Germany) with an 8-element body coil and a phased array coil. Liver protocol included morphological and functional sequences: breath-hold fat-saturated and not fat-saturated T2-weighted (T2-w) turbo spin-echo sequence, in- and opposed-phase T1-weighted (T1-w) gradient-echo sequence, dynamic imaging with a fat-saturated T1-weighted gradient-echo sequence, and diffusion-weighted imaging with echo-pulse planar sequence (EPI) at several b value 0, 50, 100, 200, 400, 600, 1000 s/mm^2^. The MR sequences were acquired in free breathing.

Detailed information regarding the MR imaging parameters is summarized in Table 2.

**Table 2:** Pulse sequence parameters on MR studies.

Sequence	Orientation	TR/TE/FA (ms/ms/deg.)	AT (min)	Acquisition Matrix	ST/Gap (mm)	FS
Trufisp T2-W	Coronal	4.30/2.15/80	0.46	512x512	4/0	without
HASTE T2-W	Axial	1500/90/170	0.36	320x320	5/0	Without and with (SPAIR)
HASTE T2w	Coronal	1500/92/170	0.38	320x320	5/0	without
SPACE T2W FS	Axial	4471/259/120	4.20	384x450	3/0	with (SPAIR)
In-Out phase T1-W	Axial	160/2.35/70	0.33	256x192	5/0	without
DWI	Axial	7500/91/90	7	192x192	3/0	with (SPAIR)
Vibe T1-W	Axial	4.80/1.76/12	0.18	320x260	3/0	with (SPAIR)

**Note.** TR = Repetition time, TE = Echo time, FA = Flip angle, AT = Acquisition time, ST = Slice thickness, FS = Fat suppression, SPAIR = Spectral adiabatic inversion recovery, HASTE = HAlf fourier Single- shot Turbo spin-Echo (HASTE), SPACE = sampling perfection with application-optimized contrasts using different flip angle evolution.

Gadoxetic acid ((0.025 mmol/kg); Primovist, Bayer Healthcare, Berlin, Germany) was injected ev at a rate of 2.0 mL/s by using a power injector (Spectris Solaris EP; Medrad, Warrendale, Pa). Arterial phase was acquired 7 s after contrast agent arrival at the thoracic aorta by using a fluoroscopic monitoring system. After contrast medium injection portal phase, transitional phase and hepatobiliary phase (HBP) were obtained 60 s, 3 minutes, and 20 minutes after, respectively.

### Images analysis

For each patient, abbreviated protocol (DWI and SPACE fat suppressed sequence) and full protocol including gadoxetic acid-enhanced MR sequence were independently and blindly assessed in random order within and between three expert radiologists with the aim of lesion detection in pre-surgical setting. The readers were blinded to previous radiological examination and pathologic results, but they were aware that the patients had colorectal cancer (CRC) and thus were at higher risk for developing metastases. To reduce recall bias, all three readers maintained an interval of more than 2 weeks between interpretation sessions of abbreviated protocol and Gd-EOB MR study. Each radiologist identified the presence of the metastasis by using the following five-point confidence scale, as we have previously defined [6]: 1 = definitely absent, 2 = probably absent, 3, equivocal, 4 = probably present, 5 = definitely present. The radiologists evaluated the following data: greatest lesion diameter, signal intensity (SI) on T1- and T2-weighted images, SI on DWI sequences and measured the ADC of each lesion, vascular enhancement pattern during arterial, portal, transitional and HBP phase for MR conventional studies. The SI of the nodule was defined as isointense, hypointense, and hyperintense compared to surrounding liver parenchyma. The diffusion-weighted signal decay was analyzed using the mono-exponential model, according to the equation ADC = (ln (S0/Sb))/b, where Sb is the signal intensity with diffusion weighting b and S0 is the non-diffusion-weighted signal intensity. This analysis was region of interest (ROI) based using median value of single voxel signals for each b value. ROIs for the tumor were manually drawn to include such hyperintense voxels on image at b value 1000 s/mm^2^. Median diffusion parameters of ROI were used as representative values for each lesion. No motion correction algorithm was used but ROIs were drawn taking care to exclude areas in which movement artifacts or blurring caused voxel misalignments. The enhancement pattern during arterial-, portal-, transitional-, and hepatobiliary phase was described as homogeneous, heterogeneous, peripheral ring enhancement, or target appearance [6]. The latter, due to the central diffusion of contrast medium, was recorded on the hepatobiliary phase images and consisted of a central area of lower degree of hypointensity compared to the periphery of the lesion [6]. In addition, the researchers were asked to record the number and segmental location of the nodule for all detected lesions.

### Statistical analysis

Data were expressed in terms of median value ± range. Detection rate of metastases on per-lesion and per-patient basis were calculated. Lesions that were assigned a grade of 4 or 5 on the confidence scale were regarded as positive for metastases and were considered to be a true-positive finding when lesion presence was pathologically confirmed. Lesions that were assigned a grade of 1 or 2 or 3 on the confidence scale were regarded as negative for metastases. We assumed a positive result for per-patient detection rate if all lesions were detected. Per-lesion detection rate was also assessed according to the pathological diagnosis and was compared between abbreviated protocol and conventional Protocol. Chi square test was performed to assess differences statistically significant among different detection rate.

Weighted к values were used to evaluate inter-reader agreement of the confidence scale regarding the presence of the lesion. к coefficients in the range of 0.81–1.0 indicated excellent agreement; those in the range of 0.61–0.80, substantial agreement; those in the range of 0.41–0.60, moderate agreement; those in the range of 0.21–0.40, fair agreement; and those in the range of 0.00–0.20, poor agreement.

A *p* value <0.05 was considered statistically significant. All analyses were performed using Statistics Toolbox of Matlab R2007a (The Math-Works Inc., Natick, USA).

## Results

The median interval between MRI and pathologic confirmation was 22 days. Lesions size ranged from 8 to 57 mm (median, 28 mm). In terms of per-lesion detection rate, all three readers had similar diagnostic detection rate with Gd-EOB MRI and with abbreviated protocol (96.8% [732 of 756] vs. 96.5% [730 of 756] for reader 1; 95.8% [725 of 756] vs. 95.2% [720 of 756] for reader 2; 96.5% [730 of 756] vs. 96.5% [730 of 756] for reader 3). Inter-reader agreement of lesions detection rate between the three radiologists was excellent (k range, 0.86–0.98) both for Gd-EOB MRI and for Fast protocol (k range, 0.89–0.99).

**Fig 2:**
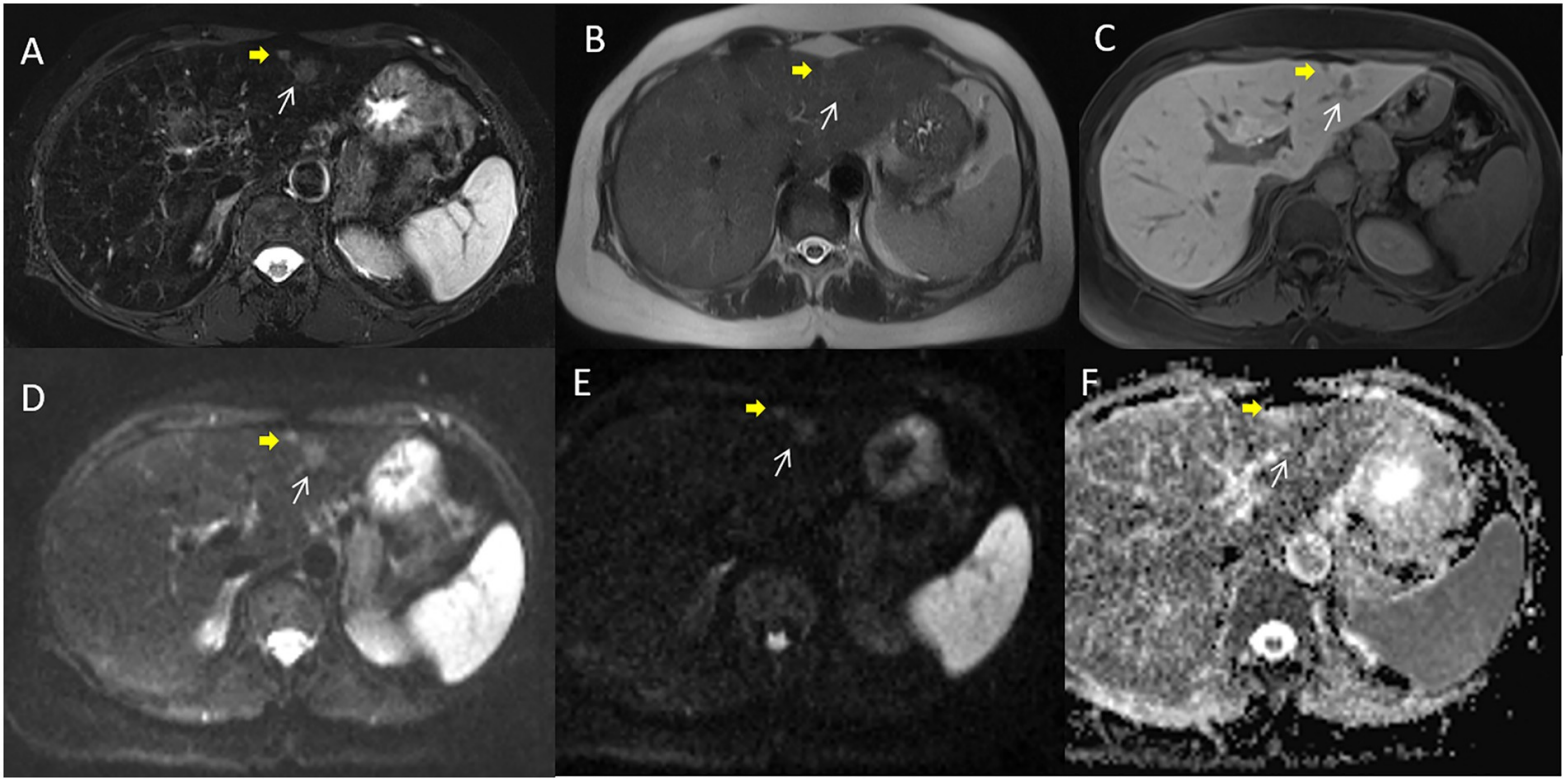
Woman 54 y with right colon cancer. Pre surgical MRI study: In A (SPACE FS T2-W sequence) white arrow shows parenchymal metastasis and yellow arrow shows subcapsular lesion. Parenchimal lesion is not detected by HAlf fourier Single- shot Turbo spin-Echo (HASTE) T2-W sequence (in B) while subcapsular lesion is detected. In EOB phase contrast study the metastases appear as hypointense lesions with restricted diffusion in DWI sequences and hypointense signal in ADC map (D, E and F).

**Fig 3:**
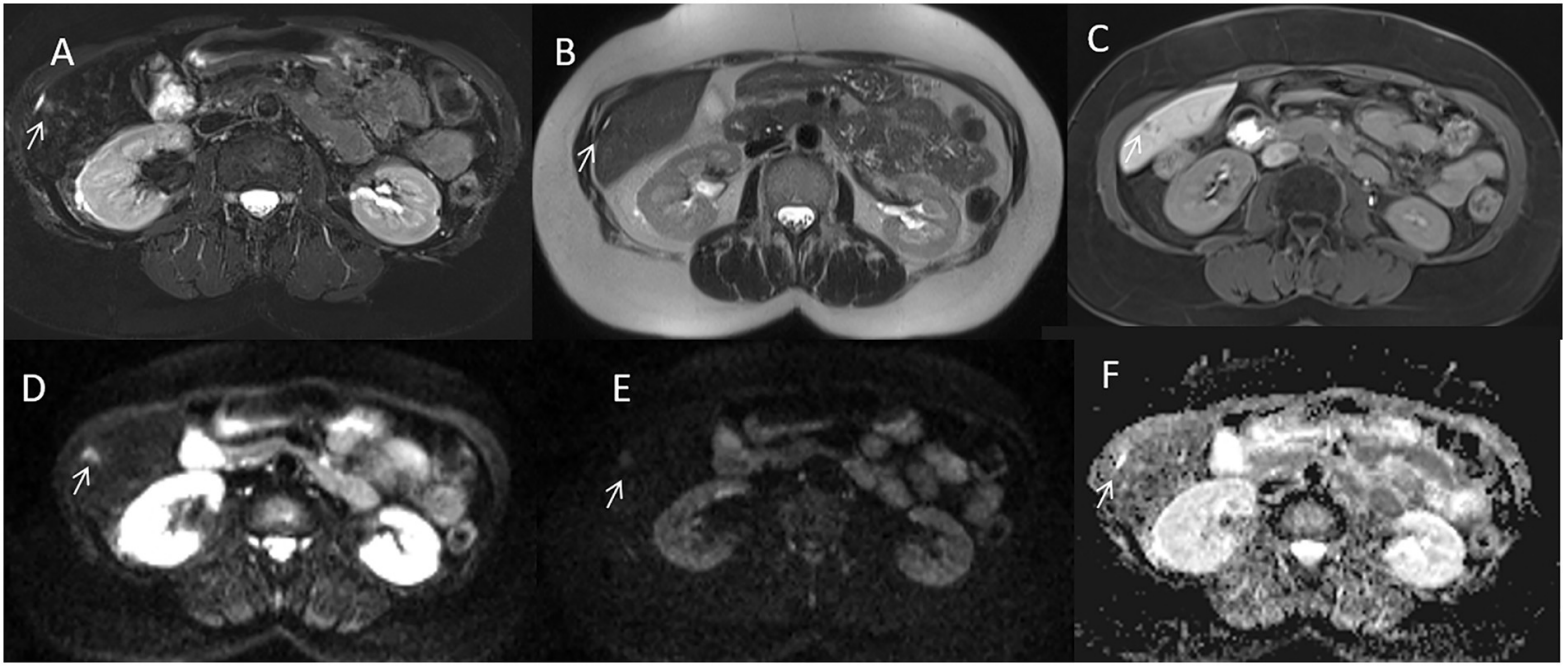
Man 63 y with rectal cancer. Pre surgical MRI study: In A (SPACE FS T2-W sequence) arrow shows subcapsular lesion. HASTE T2-W sequence (in B) does not detect the lesion, while is preset an indirect sign (capsular retraction), arrow. In EOB phase contrast study the metastasis appear as hypointense lesion with restricted diffusion in DWI sequences and hypointense signal in ADC map (D, E and F).

By consensus of three readers, the conventional MRI protocol detected 732/756 liver metastases (Fig 2) while abbreviated protocol 730/756 liver metastases (Fig 3). Nothing differences statistically was relieved between abbreviated Protocol and Conventional Protocol detection rate for each reader and for the radiological consensus obtained by three readers (p value = 0.77, 0.56, 1 and 0.77 at Chi square test, respectively).

The undetected lesions at conventional MRI protocol were: (a) 24 sub-capsular lesions (3.2%; (median diameter 9 mm; range 8–10 mm)) in 6 patients (5.6%). The additional two undetected lesions (0.3%) at abbreviated protocol were in intra-parenchymal left lobe metastases (median size 8 mm, range 8–8 mm) in 2 patients (1.9%) (Fig 3).

**Fig 4:**
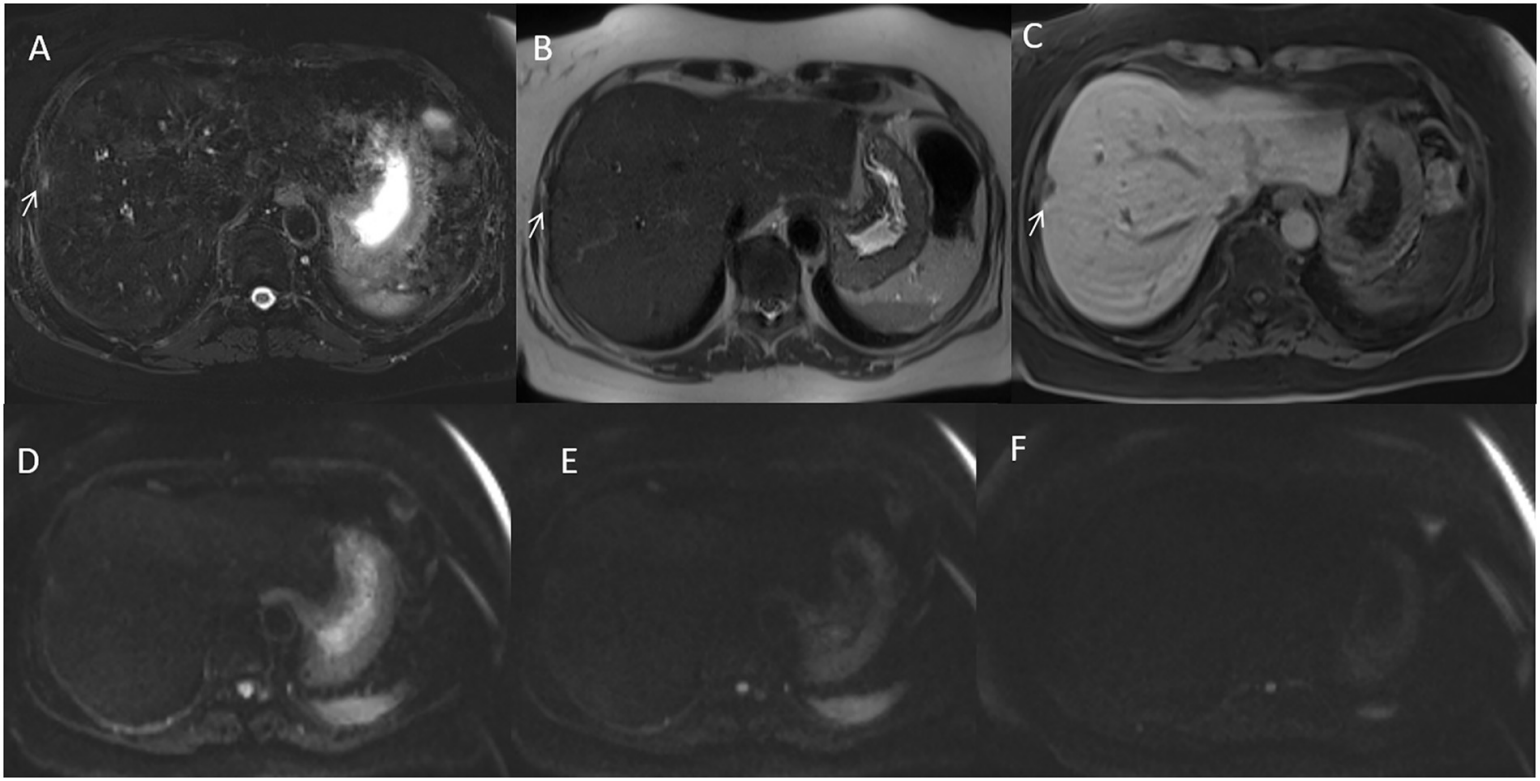
Woman 48 y with left colon cancer. Pre surgical MRI study: In A (SPACE FS T2-W sequence) arrow shows subcapsular lesion. HASTE T2-W sequence (in B) does not detect the lesion, arrow. In EOB phase contrast study the metastasis appear as hypointense lesion without restricted diffusion in DWI sequences (D, E and F).

Conventional MRI showed similar diagnostic performance compared to abbreviated protocol in per-patient detection rate, (97.2% [105 of 108] vs. 96.3% [104 of 108] for reader 1, 94.4% [102 of 108] vs. 95.4% [103 of 108] for reader 2, and 95.4% [103 of 108] vs. 94.4% [102 of 108] for reader 3). Nothing differences statistically was relieved between abbreviated Protocol and Conventional Protocol detection rate for each reader and for the radiological consensus obtained by three readers (p value = 0.70, 0.31, 0.31 and 0.45 at Chi square test, respectively) (see Fig 4).

Inter-reader agreement in per patient detection rate between the three radiologists was excellent both for Gd-EOB MRI (k range, 0.89–0.98) and for abbreviated protocol (k range, 0.90–0.99).

Imaging Features at MRI were synthesized in Table 3.

**Table 3:** Imaging features at MRI.

		Characteristic	Lesions number (%)
MR	T1-weighed sequences	homogeneously hypointense	732 (100.0%)
	T2-weighed sequences	central area of very high signal intensity	512 (69.9%)
		homogeneous hyperintense	220 (30.1%)
	DWI	restricted diffusion	732(100.0%) median ADC 1.19x10^-3^ mm^2^/s
	Gd-EOB MR Arterial phase	rim enhancement	243 (33.2%)
		hypointense	489 (66.8%)
	Gd-EOB MR Portal phase	homogeneously hypointense	732 (100%)
		enhancing rim	0 (0%)
	Gd-EOB MR transitional phase	low signal intensity compared to the surrounding parenchyma	732 (100%)
	HPB phase	homogeneously hypointense	605 (82.6%)
		target appearance	127 (17.4%)

## Discussion

Although the utility of MRI for detection and characterization of focal liver lesions is well established [1,2,3–6], its high cost and longer examination time compared with MDCT or ultrasound may limit its widespread use for staging in patients with colorectal cancer (CRC). These limits affect several oncological fields. Therefore, several recent studies investigated abbreviated and ultra-fast MRI protocols for cancer screening and diagnosis [26,27]. According to these [26,27], the present study showed that MRI acquisition time could substantially be reduced with faster, unenhanced MRI protocol maintaining the advantages of conventional protocol in detection of liver metastases. In fact, our data showed no significant difference in detection rate of gadolinium-enhanced and unenhanced MR scans for staging of liver metastases. We found a high concordance of abbreviated and conventional protocol, and using an abbreviated protocol did not result in a lower detection rate of mCRC. Although contrast enhanced imaging improved lesion detection, tissue characterization, and determination of tumor extent. However, advances in MR technology as well as improved of functional sequences as DWI give rise to the question if the administration of contrast agents is actually always needed. In fact, in pre surgical setting after conversion treatment, the radiologist’s role is identifying residual metastases in order to assess the resectability. Considering that all lesions in this phase have already been detected, we conclude that MR contrast media administration may not be necessary for pre surgical setting. Our results are similar to Tokuda and colleagues, that recently reported comparable results for differentiation of benign from malignant tumors in soft tissue masses [28] and even better results for differentiation of benign from malignant bone tumors by using fast protocol [29] suggesting that contrast enhancement is not always needed. In addition, DWI provides data about solid and cystic/necrotic tumor areas, which was previously only accessible with contrast-enhanced sequences [30]. DWI was also superior for detection of small peritoneal and serosal metastases, which were difficult to detect on gadolinium chelate enhanced images [31], it showed better contrast between tumors and bowel contents compared to gadolinium chelate enhanced images [31]. In accordance with these studies, we found similar detection rate of T2 and DW scans for lesion detection compared to conventional MR protocol.

The sensitivity in our study was higher than that recently reported by a large systematic review and meta-analysis for hepatic colorectal cancer metastasis detection [32]; in this study seventeen studies were included for analysis (from the year 1996 to 2018), comprising 1121 patients with a total of 3279 liver lesions. The pooled sensitivity, specificity, and diagnostic odds ratio were 0.90 (95% confidence intervals (CI): 0.81–0.95), 0.88 (0.80–0.92), and 62.19 (23.71–163.13), respectively. The difference in the sensitivity can be related to the absence in our population of patients without lesions However, also in this systematic review was reported that advanced scanning sequences with DWI tended to increase the sensitivity for colorectal liver metastasis detection. Hepatobiliary phase imaging can aid in lesion [32] and contrast uptake may serve a prognostic role in hepatic colorectal cancer metastases [33].

Several papers have investigated abbreviated MRI protocol in cancer detection, including many looking at HCC detection [34–41]. Lee et al [37] compared an abbreviated screening MRI protocol utilizing only dynamic contrast-enhanced images, to a conventional liver MRI (cMRI) for the characterization of observations in at-risk patients. They demonstrated that there was strong agreement between the abbreviated T1-only MRI protocol and a full liver MRI, with only 5% of cases changing LI-RADS categorization due to the inclusion of T2 and DWI. The estimated time to run this abbreviated MRI is approximately 7–10 min, possibly allowing for a more cost-effective screening MRI than our cMRIs. Marks et al. [39] evaluated the per-patient diagnostic performance of an abbreviated gadoxetic acid-enhanced MRI protocol for hepatocellular carcinoma surveillance and reported that the abbreviated MRI protocol consisting of T2-weighted and gadoxetic acid-enhanced hepatobiliary phase has high negative predictive value and may be an acceptable method for HCC surveillance while the inclusion of a DWI sequence did not significantly alter the diagnostic performance of the abbreviated protocol.

There are several features of abbreviated protocol that have to be done. First the economic implications both in terms of reduction of examination time for each patient and in terms of exam cost. The non-contract MRI examination time is approximately 8 min, therefore a reduction of 70% of time can be obtained using abbreviated protocol compared with conventional MRI. Consequently, it will be possible to study about three patients during the same time used for one patient. Also, although the exact costs of liver MRI are highly variable between different centers and countries, and it is correlated also to the contrast medium administrated; the available literature estimates a price range between to $105,02 and $3403,00 for Gd-EOB-DTPA MRI study [42–46]. As clinical implementation of fast MRI could save approximately 50% of costs. Future analyses are needed to define the cost-effectiveness of these shorter protocols. Also, there are several important features of fast protocol that facilitate clinical implementation of this technique: *(1)* intravenous access is not needed, the procedure is completely noninvasive through omitting the contrast media administration, making it suitable also for patients with impaired renal function; *(2)* patients are not exposed to risks associated with contrast administration, including allergic reactions, intracranial gadolinium deposits, and nephrogenic systemic fibrosis [17]; and *(3)* patients are required to lie motionless during the scan; therefore, a substantial shortening of acquisition time will make the MRI examination more tolerable for patients with claustrophobia and could potentially reduce motion artifacts.

Our study is not without limitations. First, the decision that lesion need to have surgical treatment was based on the conventional MRI assessment only, so that we have not evaluate the effectiveness of fast protocol in decision surgical patient management. Second, the readers involved in our study were expert readers, with an annual case load of approximately 1000 liver MRI examinations per year. Third, the MR was performed using a 1.5T MR scanner, differences in DWI acquisition between 1.5T and 3T can be present and then abbreviated MRI protocol performance for lesion detection at 3T should be demonstrated. Therefore, our results are not directly applicable to other lower-volume non expert centers or using 3T MR scanner.

## Conclusions

Abbreviated Protocol showed the same detection rate than conventional study in detection of liver metastases with a reduction of the examination time and of examination prize compared with conventional MRI. However, these results are applicable to higher-volume expert liver centers.
